# Carbapenem-Resistant Gram-Negative Bacteria in Hospitalized Patients: A Five-Year Surveillance in Italy

**DOI:** 10.3390/idr17040076

**Published:** 2025-07-02

**Authors:** Marcello Guido, Antonella Zizza, Raffaella Sedile, Milva Nuzzo, Laura Isabella Lupo, Pierfrancesco Grima

**Affiliations:** 1 Laboratory of Hygiene, Department of Biological and Environmental Sciences and Technologies, University of Salento, 73100 Lecce, Italy; marcello.guido@unisalento.it; 2 Institute of Clinical Physiology, National Research Council, 73100 Lecce, Italy; 3 Infectious Diseases Unit, Vito Fazzi Hospital, 73100 Lecce, Italy; milva.nuzzo@asl.lecce.it (M.N.); pierfrancesco.grima@asl.lecce.it (P.G.); 4 Clinical Pathology and Microbiology Laboratory, Vito Fazzi Hospital, 73100 Lecce, Italy; lauraisabella.lupo@asl.lecce.it

**Keywords:** antimicrobial resistance, Gram-negative bacteria, ESBL, carbapenem-resistant (KPC), multidrug resistance, antimicrobial stewardship, antimicrobial therapy

## Abstract

**Background/Objectives**: Antibiotic resistance is a significant and escalating challenge that limits available therapeutic options. This issue is further exacerbated by the decreasing number of new antibiotics being developed. Our study aims to describe the epidemiology and pattern of antibiotic resistance in Gram-negative infections isolated from a cohort of hospitalized patients and to analyze the distribution of infections within the hospital setting. **Methods**: A retrospective study was conducted on all patients admitted to Vito Fazzi Hospital in Lecce, Italy, who required an infectious disease consultation due to the isolation of Gram-negative bacteria from 1 January 2018 to 31 December 2022. **Results**: During the study period, 402 isolates obtained from 382 patients (240 men and 142 women) with infections caused by Gram-negative bacteria were identified. Among these isolated, 226 exhibited multidrug resistance, defined as resistance to at least one antimicrobial agent from three or more different classes. In 2018, the percentage of multidrug-resistant isolates peaked at 87.6%, before decreasing to the lowest level (66.2%) in 2021. Overall, of the 402 isolates, 154 (38.3%) displayed resistance to carbapenems, while 73 (18.1%) were resistant to extended-spectrum beta-lactamases (ESBLs). Among the resistant microorganisms, *Klebsiella pneumoniae* showed the highest resistance to carbapenems, accounting for 85.2% of all resistant strains. *Escherichia coli* exhibited the greatest resistance to ESBLs, with a rate of 86.7%. Among carbapenem-resistant *K. pneumoniae* isolates, the following resistance rates were observed: KPC-1 at 98.2%, IMP-1 at 0.9%, VIM-1 at 0.9%, and NDM-1 at 0.9%. **Conclusions**: Patients with infections caused by multidrug-resistant bacteria have limited treatment options and are therefore at an increased risk of death, complications, and longer hospital stays. Rapid diagnostic techniques and antimicrobial stewardship programs—especially for ESBLs and carbapenemases—can significantly shorten the time needed to identify the infection and initiate appropriate antimicrobial therapy compared to traditional methods. Additionally, enhancing surveillance of antimicrobial resistance within populations is crucial to address this emerging public health challenge.

## 1. Introduction

The increasing prevalence of antibiotic-resistant bacteria poses a significant threat to global health [1]. This situation emphasizes the need for a comprehensive and multidisciplinary strategy to combat the spread of resistance. Antibiotic resistance is one of the most urgent and growing challenges in healthcare, severely limiting available treatment options. This problem is exacerbated by the diminishing number of new antibiotics in the drug development pipeline [2].

In 2019, the World Health Organization (WHO) recognized antimicrobial resistance (AMR), including antibiotic resistance, as one of the top ten global health threats. This identification highlighted the urgent need for a coordinated international action plan to address the severe healthcare and economic consequences on individual and population health [3]. Antibiotic resistance can lead to treatment failure, which not only results in increased healthcare costs, but also longer hospital stays and increased mortality rates [4,5].

Drug-resistant infections result in approximately 5 million deaths each year. If we fail to take timely, effective actions against the spread of AMR, especially in low- and middle-income countries, this alarming figure could increase to 10 million deaths annually by 2050 [6].

Resistance in bacteria develops mainly through chromosomal mutations or by acquiring resistance genes. This phenomenon is commonly observed even in routine infections [7].

Particularly concerning are Gram-negative bacteria (GNB) because of their ability to mutate, acquire, and transmit plasmids and other mobile genetic elements that encode resistance genes [8]. The severity of infections caused by GNB, along with the significant public health burden they represent, adds to the urgency of this issue [9]. Notably, nine of the twelve priority bacterial pathogens identified by the WHO in 2017 are GNB, with increasingly limited treatment options due to AMR [10].

GNB can acquire genes capable of producing enzymes that inactivate antibiotics, such as extended-spectrum β-lactamases (ESBL) and carbapenemases [11,12]. GNB are frequently resistant to β-lactam antibiotics, most commonly used to treat bacterial infections. Although carbapenems are the recommended treatment for severe infections caused by ESBL-producing bacteria, cases of resistance to these antibiotics have been reported [13].

Recent data from the European Antimicrobial Resistance Surveillance Network and other low- and middle-income countries show significant variability in Carbapenem Resistance (CR) among *Klebsiella pneumoniae* (*K. pneumoniae*) isolates in hospitals.

Across European Union countries, resistance rates ranged from 0% in four countries to over 25% in six countries, with Italy reporting a resistance rate of 32.8% [12,14].

Several factors contribute to the development and spread of AMR, but the excessive use of antibiotics, including their use without proper medical indication, is considered one of the main contributors to accelerating the spread of antibiotic resistance. According to a 2017 report by the European Centre for Disease Prevention and Control (ECDC), Italy was identified as one of the countries with the most alarming levels of AMR in Europe, due to high levels of resistance in hospitals and various regions across the country [15]. In this regard, a 2021 study conducted by the ECDC and Public Health England analyzed the attitudes of European healthcare workers towards antibiotic use, revealing excessive and inappropriate use of these drugs, with an increasing trend in antibiotic prescribing and consumption [16].

Surveillance and epidemiological studies are crucial tools for preventing the severe consequences of AMR on public health. In this context, this study aims to describe the epidemiology and the antibiotic resistance pattern of GNB isolated from a cohort of patients at a hospital in southern Italy over five years, from 2018 to 2022, and analyze the distribution of these infections within the hospital environment.

## 2. Materials and Methods

### 2.1. Study Design and Subjects

A retrospective study was conducted on all patients admitted to Vito Fazzi Hospital in Lecce, Italy, for whom an infectious disease consultation was requested due to gram-negative isolates and antimicrobial resistance profiles between 1 January 2018 and 31 December 2022.

Patient records were collected, including age, sex, years, operative clinical unit, biological sample, and identification of medically relevant Gram-negative and antimicrobial resistance.

Duplicate microbiological tests from the same patient, tests performed within the first 48 h of hospitalization, and tests from patients readmitted within 3 days were excluded from the sample. Records with incomplete data were excluded from the analysis.

The study was conducted in compliance with the Declaration of Helsinki and was approved by the Regional Ethics Committee (Minutes No. 10 of 14 December 2022). Due to the retrospective nature of the study, informed consent was not necessary.

### 2.2. Specimen Processing and Identification

The clinical specimens were processed, and the bacteria identified using VITEK MS (bioMérieux, Marcy l’Etoile, France), a MALDI-TOF-based identification system, at the Clinical Pathology and Microbiology Unit of the Hospital. Standard operating procedures for specimen processing and culture in the laboratory were strictly followed. The microorganisms were then analyzed by the VITEK MS automated system using a mass spectrometry method with estimation of ribosomal protein profiles.

For *E. coli*, the ATCC 25922 standard QC strain was run alongside clinical samples to monitor VITEK performance [17].

Clinical specimens included sputum, bronchoalveolar lavage (BAL), bronchial aspirate (BAS), pleural effusion (PE), surgical wound, cerebrospinal fluid (CSF), bone, blood, rectal swab, and urine.

### 2.3. Antimicrobial Susceptibility Testing

The susceptibility of clinically significant GNB to antimicrobial agents was performed with the Vitek 2 system (bioMérieux, France). Antibiotic resistance was detected to determine the minimum inhibitory concentration (MIC) for antibiotics such as Amikacin, Gentamicin, Amoxicillin/Clavulanic acid, Piperacillin/Tazobactam, Cefepime, Ceftazidime, Ceftazidime/Avibactam, Ceftolozane/Tazobactam, Ciprofloxacin, Ertapenem, Imipenem, Meropenem, Fosfomycin, Tigecycline, and Trimethoprim/Sulfamethoxazole.

The molecules actually responsible for the targeted therapy or the reference ones have been included in the antibiogram profiles.

Results were interpreted according to the clinical breakpoints of the European Union Committee on Antimicrobial Susceptibility Testing, EUCAST v.13.0 [18].

Four variables were used to identify AMR status: (1) AMR defined as resistance to at least one antimicrobial agent; (2) MDR (Multidrug Resistance) defined as resistance to at least one antimicrobial in three or more antimicrobial categories; (3) XDR (Extended Drug Resistance) defined as resistance to at least one antimicrobial in all but one or two classes and (4) CR defined as resistance to at least one carbapenem antibiotic [19]. All CR-GNB were screened using the Xpert Carba-R assay, a commercial kit designed for use with GeneXpert^®^ Instrument systems.

This multiple qualitative in vitro diagnostic test is designed to detect and differentiate the gene sequences associated with carbapenem-non-susceptibility, including bla_KPC_, bla_NDM_, bla_VIM_, bla_OXA-48_, and bla_IMP_. The test employed automated real-time polymerase chain reaction (PCR) technology.

### 2.4. Statistical Analysis

Data are presented as the number of isolates and their percentages. Continuous variables were expressed as mean ± standard deviation (SD), while categorical variables were presented as frequency and percentage. Levene’s test is used to assess the homogeneity of the data. Comparisons between continuous variables are performed using a two-tailed unpaired *t*-test, and comparisons between categorical variables are conducted using the chi-square test or Fisher’s exact test, as appropriate. The logistic regression was used to evaluate temporal trends and multiple comparisons of antibiotics. The false-discovery rate method of Benjamini-Hochberg was used for the correction of antibiotic multiple comparisons. A *p*-value of less than 0.05 is considered statistically significant.

All statistical analyses were performed using the SPSS (Statistical Package for Social Sciences) software version 24.0 (IBM Corp., Armonk, NY, USA).

## 3. Results

During the study period, 402 isolates obtained from 382 patients (240 males and 142 females) with infections caused by GNB were identified. The mean age of the subjects was 68.6 ± 15.4 years. The primary age group affected by the infection was subjects over 65 years (67.5%), followed by adults aged 46 to 65 years (23.6%).

The trend by year of patients showed a decrease between 2018 and 2020 and a subsequent increase between 2020 and 2022, with the highest peak observed in 2022 with 126 isolates (Table 1).

**Figure 1:**
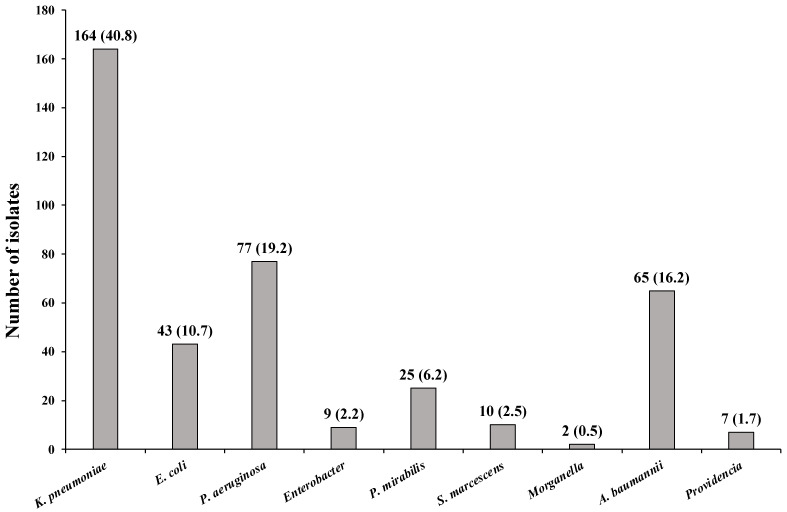
Number and frequency of microorganisms isolated from the patient.

Among the isolated microorganisms, *K. pneumoniae* was the most frequent pathogen, with 164 (40.8%) isolations, followed by *Pseudomonas aeruginosa* (*P. aeruginosa*) at 77 (19.15%), *Acinetobacter baumannii* (*A. baumannii*) at 65 (16.17%), and *Escherichia coli* (*E. coli*) at 43 (10.7%) (Figure 1).

**Figure 2:**
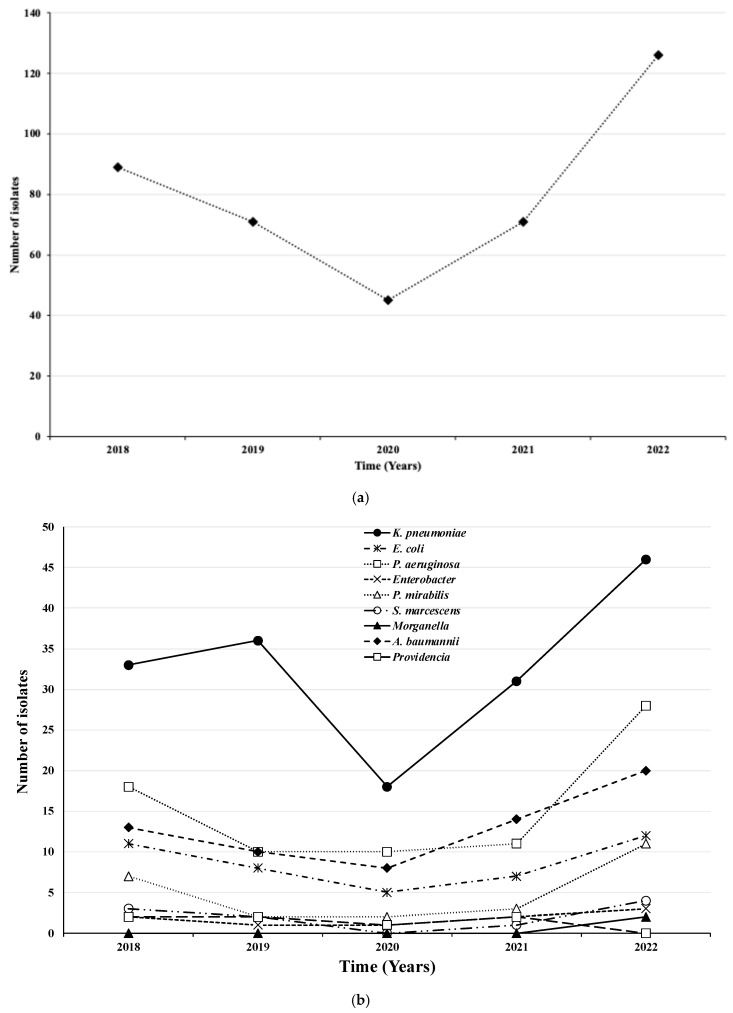
Total number (**a**) and type (**b**) of microorganisms isolated by year.

In 2020, the lowest number of isolates was observed for all microorganisms, while the highest values were recorded in 2022 (Figure 2a). The highest numbers of isolates were found for *K. pneumoniae* (*n* = 46), *P. aeruginosa* (*n* = 28), and *A. baumannii* (*n* = 20) (Figure 2b).

Table 2 reports the prevalence of microorganisms isolated from the different biological samples. Specifically, the total row reports the number of isolates detected for each biological sample and the percentage of the total isolates (*n* = 402), and each row reports the number of each microorganism isolated in the specific biological sample, and in brackets, the percentage of the total isolates for each microorganism is reported.

135 (33.6%) isolates were from urine, 83 (20.6%) from BAS, 90 (22.4%) from blood, 45 (11.2%) from surgical wounds, 17 (4.2%) from sputum, 13 (3.2%) from BAL, 12 (3%) from rectal swab, 3 (0.8%) from bone and pleural fluid, and 1 (0.2) from cerebrospinal fluid.

Table 3 reports the microorganisms isolated in the different Clinical Operative Units. Specifically, the total column shows the total number of microorganisms in the different units and the percentage of the total of isolates (*n* = 402).

The number of each microorganism isolated in the operative unit is reported in the rows, and the percentage of the total number of each microorganism isolated is reported in brackets. In 19 different Operating Units, the highest number of isolates was recorded in the General Medicine department, with 84 isolates (20.9%). This was followed by the Intensive Care Unit with 68 isolates (16.9%) and both departments of Cardiac Surgery and Orthopedics with 46 isolates (11.4%). In contrast, the lowest number of isolates was found in the Gynecology and Ophthalmology Units, with only one isolate (0.2%) in each department.

In particular, *K. pneumoniae* and *P. aeruginosa* were mainly isolated in the General Medicine department, with 41 (25.0%) and 17 (22.1%) microorganisms isolated, respectively. *A. baumannii* was instead primarily isolated in the Intensive Care Unit with 29 (44.6% isolated) and *E. coli* in Orthopedics with 12 (27.9%) isolates (Table 3).

The AMR patterns are shown in Table 4. Among the antibiotics tested, the highest resistance was found to Ciprofloxacin with 68.8% of resistant isolates, followed by Trimethoprim/sulfamethoxazole (65.6%), Ceftazidime (60.8%), and Amoxicillin/Clavulanic acid (60.0%). Ceftazidime/Avibactam are the antibiotics with the lowest resistance patterns, with percentages of 18.1% and 18.4%, respectively.

Table 5 shows, by year, the percentage of isolates resistant to each antimicrobial agent and the number of isolates tested in parentheses. AMR trends from 2018 to 2022 indicated the lowest levels of resistance in 2020 and 2021 across all antibiotic classes, with significant differences observed between years for Aminoglycosides (*p* = 0.0299), Beta-Lactams (*p* = 0.0270c), Fluoroquinolones (*p* = 0.0185), Glycylcycline (*p* = 0.0240), and Sulfonamides (*p* = 0.0097) (Table 5).

Among the various classes of antibiotics, carbapenems exhibited higher levels of resistance during the study period, except in 2019, when the highest level of resistance was recorded for beta-lactams, reaching 62.9%.

Overall, 85.1% (342/402) of the collected samples showed AMR, and 65.4% (263/402) MDR. MDR was found in 87.6% of isolates in 2018; the lowest levels were instead observed in 2021 in 47.6% of isolated samples. Additionally, XDR was observed in a small percentage of samples; 3.2% (13/402) showed resistance to 14 of the 16 antimicrobials tested.

Overall, AMR, MDR, and XDR showed decreasing temporal trends with significant differences (*p* = 0.0018, *p* < 0.0001, and *p* < 0.0001, respectively) (Table 5).

Overall, AMR, MDR, and XDR showed decreasing temporal trends with significant differences between years (*p* = 0.0018, *p* < 0.0001, and *p* < 0.0001, respectively) (Table 5).

Significant differences were observed in the temporal trend of 8 out of 16 antimicrobials, covering five of the nine drug classes evaluated. Isolates showed significant decreasing temporal trends in resistance to amikacin (*p* = 0.0024), amoxicillin/clavulanic acid (*p* < 0.0001), and trimethoprim/sulfamethoxazole (*p* = 0.0097). Annual changes in resistance to ertapenem and tigecycline are difficult to assess as data for 2021 and 2022 are not available. A reversal of the decreasing trend was observed in the last year for ceftazidime (*p* = 0.0006) and ciprofloxacin (*p* = 0.0185) (Table 5).

The highest percentage of resistance among antibiotics was recorded for ciprofloxacin (68.8%; 273/397), trimethoprim/sulfamethoxazole (65.6%; 227/346), ceftazidine (60.8%; 203/334), and amoxicillin/clavulanic acid (60.0%; 75/125) (Table 5). *K. pneumoniae*, *A. baumannii*, and *P. aeruginosa* are the microorganisms that have developed the greatest resistance to these four antibiotics.

In particular, among the microorganisms resistant to ciprofloxacin, *K. pneumoniae* (50.5%; 138/273), *A. baumannii* (21.2%; 59/273), and *P. aeruginosa* (11.4%; 31/273) are the most widespread. Among the microorganisms resistant to Trimethoprim/Sulfamethoxazole, *K. pneumoniae* (48.2%; 79/164) is the most common, followed by *A. baumannii* (22.6%; 37/164) and *P. aeruginosa* (12.2%; 20/164). Among the microorganisms resistant to Ceftazidime, *K. pneumoniae* (50.7%; 73/144) is the most common, followed by *A. baumannii* (25.0%; 36/144) and *P. aeruginosa* (11.1%; 20/144). Finally, among the microorganisms resistant to Amoxicillin/Clavulanic acid, *K. pneumoniae* (53.6%; 81/151) is the most common, followed by *A. baumannii* (21.2%; 32/151) and *P. aeruginosa* (9.9%; 15/151).

Overall, 154 (38.3%) of the 402 isolates showed resistance to Carbapenems and 73 (18.1%) showed resistance to the ESBL-producing organism. Among the resistant microorganisms, *K. pneumoniae* was the most resistant to Carbapenems, with a percentage equal to 85.2% of the total resistant ones, while *E. coli* was the most resistant to ESBL, with a rate equal to 86.7% of the resistant microorganisms. (Table 6). The isolates resistant to any carbapenem were stratified by gene. Among the carbapenem-resistant isolates of *K. pneumoniae*, *KPC-1*, *IMP-1*, *VIM-1*, and *NDM-1* were detected in 98.2%, 0.9%, 0.9%, and 0.9% of the isolates, respectively (Table 7).

## 4. Discussion

Understanding the distribution of pathogens and monitoring antibiotic resistance is essential for controlling infectious diseases and implementing effective treatment strategies.

This study aims to investigate the distribution of Gram-negative bacteria in our hospital from 2018 to 2022 and the antibiotic resistance patterns among the isolated bacteria.

In total, 402 bacteria were isolated during this research. Among these, *K. pneumoniae* accounted for 40.8% of the isolates, making it the most frequent pathogen, followed by *P. aeruginosa* at 19.15%, *A. baumannii* at 16.17%, and *E. coli* at 10.7%.

According to data from the ECDC in 2023, based on 309,504 patients across 1332 hospitals, the most frequently isolated Gram-negative bacteria in hospitals throughout the EU and EEA were *E. coli* and *Klebsiella* spp. (of which, 78.3% were *K. pneumoniae*), *P. aeruginosa*, *Proteus* spp., *Acinetobacter* spp., and *Enterobacter* spp. Notable differences in isolation percentages were observed between countries. In Italy in 2023, *Klebsiella* spp. was reported as the most frequently isolated Gram-negative bacteria, and our findings are consistent with these data [14].

In our research, we found that the most vulnerable group to bacterial infections is primarily male individuals, who make up 62.8% of cases.

The literature data indicate that males are more susceptible to infections than females [20] and have a higher mortality rate associated with these infections [21]. The higher mortality rate in males is generally attributed to infections caused by antimicrobial-resistant pathogens. Supporting this, a recent study conducted in Southern Italy found that 60% of patients with bloodstream infections caused by carbapenem-resistant *K. pneumoniae* were male [22].

There is substantial evidence suggesting that the increased susceptibility, prevalence, and severity of infections in males result from both weaker innate and adaptive immune responses [23] and from differences in health-seeking behavior, quality of healthcare received, and adherence to treatment recommendations [24].

However, some studies have reported a uniform distribution between males and females [25] or in some cases, a higher prevalence of infections among females compared to males [26].

Most of these cases occurred among individuals over the age of 65 years, representing 67.5% of the affected population. This result aligns with a Spanish study, in which 66.9% of the samples collected were from patients over 60 years of age [20].

Furthermore, we observed a decreasing trend in infection rates, followed by an increase in the subsequent years of 2021–2022.

The data from our study correspond to the time period surrounding the COVID-19 pandemic. Changes in human behavior during 2020 and 2021, driven by efforts to control the spread of the virus, have influenced the risk of infection with AMR pathogens [27,28]. Since 2021, as social distancing measures have gradually been lifted, a noticeable increase in infections has been observed.

The highest percentage of microorganisms was isolated from urine and blood samples, while a lower percentage was obtained from respiratory samples (BAS, BAL, PE, sputum) and various other clinical samples, such as surgical wounds, cerebrospinal fluid, bones and rectal swabs.

Many similar studies have focused primarily on bacterial infections in the bloodstream [22,29,30]. In contrast, our research highlighted the spread of bacterial infections, particularly by urinary isolates, which are among the most common indications for antibiotic prescription, and significant contributors to antimicrobial resistance, driving local stewardship decisions [31].

Among the main microorganisms isolated, *A. baumannii* was primarily found in respiratory samples, representing 52.3% of the total. In contrast, *E. coli* and *K. pneumoniae* were predominantly isolated from urine samples, accounting for 65.1% and 39%, respectively. Our results are consistent with those reported by Morales et al. in a study conducted in 2024 [20] that highlighted a significant presence of bacterial isolates in areas outside the bloodstream, underscoring the necessity for effective monitoring and surveillance of bacterial infections in healthcare environments.

The highest number of pathogens was isolated from the General Medicine department, followed by the Intensive Care Unit (ICU). The Cardiac Surgery and Orthopedics wards had an equal percentage of isolated pathogens. These data reveal a higher prevalence of GNB infections in surgical departments and the ICU, which aligns with existing literature on the subject [32].

Most of the isolates exhibited high resistance to ciprofloxacin, trimethoprim/sulfamethoxazole, ceftazidime, and amoxicillin/clavulanic acid. In contrast, the majority of the isolates were found to be sensitive to ceftazidime/avibactam.

These findings highlight the importance of epidemiological studies for implementing appropriate empiric therapy for hospitalized patients. Research has shown that the likelihood of providing effective empirical antibiotic treatments is significantly higher when guidelines based on local residence patterns are followed [33].

Moreover, we observed a high prevalence of resistance to meropenem. Our results confirm European data that place Italy among the countries with the highest rates in Europe, with a percentage of resistant strains around 24.9% [14]. This finding supports the need to develop a carbapenem-sparing program when selecting antibiotic regimens in hospital settings.

Our results indicate that from 2019 to 2021, there was a significant reduction in the prevalence of MDR strains, followed by an initial increase. This trend can be partially explained by the peak of the SARS-CoV-2 pandemic during that period. The drastic reduction in hospital admissions unrelated to COVID-19 led to a decrease in antibiotic prescriptions. After the pandemic emergency subsided, we observed a rise in antibiotic prescriptions, which coincided with a slight increase in the prevalence of MDR bacteria. This observation confirms the importance of implementing a meticulous stewardship program tailored to local epidemiology and the need for ongoing updates of epidemiological data.

Additionally, the high prevalence of MDR isolates is concerning, particularly the significant resistance observed in *K. pneumoniae* (82.3%) and *P. aeruginosa* (70.1%) to the tested antibiotics. Notably, 85.2% of *K. pneumoniae* and 51.9% of *P. aeruginosa* showed resistance to carbapenems. This data reinforces the recommendation for using rapid diagnostic techniques instead of standard microbiological tests, as these can reduce the time between infection onset and the initiation of appropriate antimicrobial therapy in septic patients.

Patients with infections caused by MDR bacteria face limited treatment options, which increases their risk of death, complications, and prolonged hospital stay. For Gram-negative pathogens, especially in the case of ESBL and carbapenemase-producing strains, the adoption of rapid diagnostic techniques combined with antimicrobial stewardship programs significantly reduces the time needed for identification of microorganisms compared to traditional methods. The capacity of rapid tests to quickly identify a wide variety of pathogens and their antibiotic resistance offers the opportunity for the early initiation of targeted antibiotic therapy, thus avoiding the ineffective and excessive use of antibiotics [34].

A retrospective study conducted by Walker et al. [35] also showed that 30-day mortality was significantly lower following the introduction of rapid tests.

Effective antibiotic management and quick detection of drug resistance are crucial in reducing the spread of drug-resistant pathogens. However, enhancing AMR surveillance within populations remains a key measure for addressing this emerging public health challenge [36].

Our study has several limitations. First, as this is a retrospective study, we exclusively focused on data obtained from microbiology laboratory records of hospitalized patients with positive clinical cultures for gram-negative bacteria, excluding the investigation of the transmission pattern.

Furthermore, this data included only basic information such as patient sex, date of hospitalization, type of specimen tested, and antimicrobial susceptibility test results.

The lack of specific clinical data, such as co-morbidities, prior antibiotic exposure, invasive procedures and length of hospital stay, restricts our ability to identify and analyze risk factors contributing to antibiotic-resistant GNB.

The prevalence of Gram-positive bacteria and the related antibiotic susceptibility have not been assessed, but would be useful to plan the evaluation for an accurate stewardship program.

Additionally, our study was conducted in a single hospital in Italy with a small sample size, which limits the generalizability of the results to other Italian hospitals. Therefore, future large-scale, multicenter studies at the national level should be conducted among patients infected with GNB to determine the local prevalence of antibiotic-resistant GNB and to identify associated risk factors.

Furthermore, it would be desirable to use genomic sequencing methods to precisely identify the genes and mutations responsible for antibiotic resistance, track the spread of resistant strains, and promote a personalized approach to the clinical management of infections.

Despite these limitations, this study has heightened awareness of common microorganisms identified in our region, their distributions, and their antibiotic resistance. This information could help healthcare professionals and policymakers make informed decisions regarding patient management.

## 5. Conclusions

The continuous evolution of resistance and its spread among bacteria, leading to decreased effectiveness of antibiotics, presents major challenges for clinical practice and public health, necessitating global action.

Data regarding the spread of antibiotic resistance in these pathogens may help control the transmission of such infections.

In our study, about 40% of isolates showed resistance to carbapenems and nearly 20% exhibited resistance to ESBL. A significant number of *K. pneumoniae* and *P. aeruginosa* isolates were found to be resistant to the antibiotics tested. These resistances contribute to increased mortality rates and present a challenge for physicians and healthcare professionals when treating hospital-acquired or community-acquired infections. Our research highlights a critical issue that is not widely recognized in our reality. As a result, it emphasized the need for further studies to better understand the problem and to develop improved infection control strategies and antibiotic management, particularly in specific clinical settings such as certain hospital wards.

## Figures and Tables

**Table 1:** Demographic characteristics of study patients (*n* = 382).

Parameters	Female (*n* = 142)	Male (*n* = 240)	Total (*n* = 382)	*p*
Age, mean ± SD	69.4 ± 14.6	68.1 ± 15.9	68.6 ± 15.4	0.206 ^1^
Age group				
1–25, *n* (%)	3 (2.1)	5 (2.1)	8 (2.1)	
26–45, *n* (%)	5 (3.5)	21 (8.7)	26 (6.8)	
46–65, *n* (%)	43 (30.3)	47 (19.6)	90 (23.67)	
>65, *n* (%)	91 (64.1)	167 (69.6)	258 (67.5)	0.040 ^2^
Years				
2018, *n* (%)	31 (21.8)	51 (21.2)	82(21.5)	
2019, *n* (%)	27(19.0)	39 (16.3)	66 (17.3)	
2020, *n* (%)	16 (11.3)	23 (9.6)	39 (10.2)	
2021, *n* (%)	18 (12.7)	53 (22.1)	71 (18.6)	
2022, *n* (%)	50 (35.2)	74 (30.8)	124 (32.4)	0.246 ^3^

^1^ Two-tailed unpaired Student *t*-test; ^2^ Fisher’s exact test; ^3^ Chi-square test.

**Table 2:** Prevalence of microorganisms isolated from the indicated biological samples.

Microbe	Sputum *n* (%)	BAL *n* (%)	BAS *n* (%)	PE *n* (%)	Surgical Wound *n* (%)	CSF *n* (%)	Bone *n* (%)	Blood *n* (%)	Rectal Swab *n* (%)	Urine *n* (%)
*K. pneumoniae*	6 (3.7)	2 (1.2)	24 (14.6)	1 (0.6)	16 (9.8)	1 (0.6)	-	39 (23.8)	11 (6.7)	64 (39.0)
*E. coli*	-	1 (2.3)	1 (2.3)	1 (2.3)	3 (7.0)	-	-	8 (18.6)	1 (2.3)	28 (65.1)
*P. aeruginosa*	4 (5.2)	4 (5.2)	18 (23.4)	1 (1.3)	10 (13.0)	-	2 (2.6)	19 (24.7)	-	19 (24.7)
*E. cloacae*	-	1 (11.1)	2 (22.2)	-	4 (44.4)	-	-	1 (11.1)	-	1 (11.1)
*P. mirabilis*	1 (4.0)	-	6 (24.0)	-	6 (24.0)	-	1 (4.0)	-	-	11 (44.0)
*S. marcescens*	1 (10.0)	-	6 (60.0)	-	-	-	-	2 (20.0)	-	1 (10.0)
*M. morganii*	-	-	-	-	-	-	-	1 (50.0)	-	1 (50.0)
*A. baumannii*	5 (7.7)	5 (7.7)	24 (36.9)	-	5 (7.7)	-	-	19 (29.2)	-	7 (10.8)
*P. stuartii*	-	-	2 (28.6)	-	1 (14.3)	-	-	1 (14.3)	-	3 (42.9)
Total	17 (4.2)	13 (3.2)	83 (20.6)	3 (0.8)	45 (11.2)	1 (0.2)	3 (0.8)	90 (22.4)	12 (3.0)	135 (33.6)

BAL—Bronchoalveolar lavage; BAS—Bronchial Aspirate; PE—Pleural Effusion; CSF—Cerebrospinal fluid.

**Table 3:** Isolated microorganisms in the different operative clinical units.

Clinical Operative Units	*Klebsiella* *pneumoniae* *n* (%)	*Escherichia* *coli* *n* (%)	*Pseudomonas* *aeruginosa* *n* (%)	*Enterobacter* *cloacae* *n* (%)	*Proteus* *mirabilis* *n* (%)	*Serratia* *marcescens* *n* (%)	*Morganella* *morganii* *n* (%)	*Acinetobacter baumannii* *n* (%)	*Providencia* *stuartii* *n* (%)	Total *n* (%)
Cardiac surgery	20 (12.2)	6 (14.0)	8 (10.4)	3 (33.3)	5 (20.0)	1 (10.0)	- (-)	3 (4.6)	- (-)	46 (11.4)
Cardiology	14 (8.5)	5 (11.6)	5 (6.5)	1 (11.1)	3 (12.0)	2 (20.0)	- (-)	2 (3.1)	- (-)	32 (8.0)
General surgery	2 (1.2)	1 (2.3)	1 (1.3)	1 (11.1)	- (-)	- (-)	- (-)	1 (1.5)	- (-)	6 (1.5)
Thoracic surgery	2 (1.2)	1 (2.3)	1 (1.3)	- (-)	- (-)	- (-)	- (-)	- (-)	- (-)	4 (1.0)
Hematology	5 (3.0)	- (-)	7 (9.1)	- (-)	- (-)	- (-)	- (-)	- (-)	- (-)	12 (3.0)
Gynecology	- (-)	- (-)	1 (1.3)	- (-)	- (-)	- (-)	- (-)	- (-)	- (-)	1 (0.2)
General medicine	41 (25.0)	3 (7.0)	17 (22.1)	- (-)	5 (20.0)	3 (30.0)	- (-)	13 (20.0)	2 (28.6)	84 (20.9)
Nephrology	11 (6.7)	2 (4.7)	2 (2.6)	- (-)	1 (4.0)	- (-)	- (-)	1 (1.5)	- (-)	17 (4.2)
Neurology	2 (1.2)	1 (2.3)	2 (2.6)	- (-)	2 (8.0)	1 (10.0)	- (-)	2 (3.1)	- (-)	8 (2.0)
Neurosurgery	10 (6.1)	- (-)	8 (10.4)	2 (22.2)	- (-)	1 (10.0)	- (-)	3 (4.6)	1 (14.3)	27 (6.7)
Ophthalmology	- (-)	- (-)	- (-)	- (-)	1 (4.0)	- (-)	- (-)	- (-)	- (-)	1 (0.2)
Oncology	2 (1.2)	- (-)	1 (1.3)	- (-)	- (-)	- (-)	- (-)	- (-)	- (-)	3 (0.7)
Orthopedics	15 (9.1)	12 (27.9)	1 (1.3)	- (-)	3 (12.0)	- (-)	- (-)	3 (4.6)	- (-)	46 (11.4)
Pneumology	7 (4.3)	1 (2.3)	13 (16.9)	- (-)	3 (12.0)	1 (10.0)	- (-)	8 (12.3)	2 (28.6)	26 (6.5)
Psychiatry	- (-)	2 (4,7)	1 (1.3)	- (-)	- (-)	- (-)	- (-)	- (-)	- (-)	3 (0.7)
Intensive care	25 (15.2)	2 (4.7)	4 (5.2)	2 (22.2)	1 (4.0)	1 (10.0)	2 (100)	29 (44.6)	2 (28.6)	68 (16.9)
Urology	8 (4.9)	7 (16.3)	2 (2.6)	- (-)	1 (4.0)	- (-)	- (-)	- (-)	- (-)	18 (4.5)

**Table 4:** Overall antimicrobial resistance pattern of bacteria isolated from different clinical specimens.

Antimicrobial Agents	No. of Bacteria Tested	Susceptibility Patterns		
		Resistant No. (%)	Intermediate No. (%)	Sensitive No. (%)
Amikacin	335	103 (30.7)	9 (2.7)	223 (66.6)
Amoxicillin/Clavulanic acid	125	75 (60.0)	- (-)	50 (40.0)
Cefepime	243	126 (51.9)	42 (17.3)	75 (30.9)
Ceftazidime	334	203 (60.8)	36 (10.8)	95 (28.4)
Ceftazidime/Avibactam	182	33 (18.1)	- (-)	149 (81.9)
Ceftolozane/Tazobactam	182	79 (43.4)	- (-)	103 (56.6)
Ciprofloxacin	397	273 (68.8)	35 (8.8)	89 (22.4)
Ertapenem	139	79 (56.8)	2 (1.4)	79 (56.8)
Fosfomycin	155	88 (56.8)	- (-)	67 (43.2)
Gentamicin	351	147 (41.9)	8 (2.3)	147 (41.9)
Imipenem	279	144 (51.6)	50 (17.9)	85 (30.5)
Meropenem	401	209 (52.1)	11 (2.7)	181 (45.1)
Piperacillin/Tazobactam	334	188 (56.3)	34 (10.2)	112 (33.5)
Tigecycline	82	36 (43.9)	23 (28.0)	23 (28.0)
Trimethoprim/Sulfamethoxazole	346	227 (65.6)	2 (0.6)	117 (33.8)

**Table 5:** Trends in antimicrobial resistance of resistant isolates to antimicrobial agents, 2018–2022.

Group/Antimicrobial	2018 % (*n*)	2019 % (*n*)	2020 % (*n*)	2021 % (*n*)	2022 % (*n*)	Total % (*n*)	*p* *°
**Aminoglycosides**	**61.8 (89)**	**56.3 (40)**	**40.0 (45)**	**47.9 (34)**	**34.1 (43)**	**52.1 (251)**	**0.0019**
Amikacin	36.0 (82)	38.0 (71)	29.7 (37)	27.5 (51)	20.2 (94)	30.7 (335)	0.0018
Gentamicin	46.1 (89)	38.0 (71)	33.3 (39)	43.1 (58)	43.6 (94)	41.9 (351)	0.0017
**Beta-Lactams**	**73.7 (76)**	**67.2 (61)**	**56.8 (37)**	**60.7 (56)**	**58.1 (105)**	**63.6 (335)**	**0.0915**
Amoxicillin/Clavulanic acid	80.4 (56)	73.7 (19)	45.5 (11)	33.3 (15)	25.0 (24)	60.0 (125)	0.0016
Piperacillin/Tazobactam	61.8 (76)	57.4 (61)	48.6 (37)	51.8 (56)	57.1 (105)	56.4 (335)	0.6240
**Cephalosporins**	**58.4 (89)**	**65.0 (60)**	**51.4 (37)**	**54.4 (57)**	**63.8 (105)**	**62.1 (348)**	**1.0500**
Cefepime	60.0 (55)	20.0 (5)	45.5 (22)	50.9 (57)	51.0 (104)	51.9 (243)	1.3212
Ceftazidime	69.3 (76)	65.0 (60)	51.4 (37)	52.6 (57)	60.0 (105)	60.8 (335)	0.5220
Ceftazidime/Avibactam	- (0) ^§^	- (0) ^§^	30.0 (20)	14.0 (57)	18.1 (105)	18.1 (182)	1.2988
Ceftolozane/Tazobactam	- (0) ^§^	- (0) ^§^	42.9 (21)	40.4 (57)	45.2 (104)	43.4 (182)	1.3705
**Fluoroquinolones**							
Ciprofloxacin	77.5 (89)	72.9 (52)	68.9 (45)	60.6 (71)	64.8 (122)	68.8 (379)	0.5797
**Carbapenems**	**66.3 (89)**	**56.3 (71)**	**53.3 (45)**	**54.9 (71)**	**53.6 (125)**	**57.1 (401)**	**1.2296**
Ertapenem	61.8 (76)	52.9 (51)	41.7 (12)	- (0) ^§^	- (0) ^§^	56.8 (139)	1.1172
Imipenem	45.2 (62)	0.0 (4)	56.5 (23)	54.3 (70)	54.2 (120)	51.6 (279)	0.3416
Meropenem	53.9 (89)	56.3 (71)	48.9 (45)	54.9 (71)	48.0 (125)	52.1 (401)	1.0548
**Phosphonics**							
Fosfomycin	62.5 (88)	48.1 (54)	50.0 (12)	- (0)	100 (1)	56.8 (155)	0.9014
**Glycylcyclines**							
Tigecycline	44.8 (58)	57.1 (14)	10.5 (19)	- (0) ^§^	- (0) ^§^	43.9 (91)	1.2348
**Sulfonamides**							
Trimethoprim/Sulfamethoxazole	75.3 (89)	69.8 (63)	58.3 (36)	63.9 (61)	57.7 (97)	65.6 (346)	0.5135
**AMR**	**96.6 (89)**	**85.9 (71)**	**80.0 (45)**	**80.3 (71)**	**81.0 (126)**	**85.1 (402)**	**0.0018**
**MDR**	**87.6 (89)**	**74.6 (71)**	**62.2 (45)**	**62.0 (71)**	**47.6 (126)**	**65.4 (402)**	**<0.0001**
**XDR**	**10.1 (89)**	**4.2 (71)**	**2.2 (45)**	**0.0 (71)**	**0.0 (126)**	**3.2 (402)**	**<0.0001**

* Logisitic regression; AMR—Antimicrobial Resistance; MDR—Multidrug Resistance; XDR—Extensive drug resistance. °—Every *p*-value except the last three has been corrected for multiple comparisons; ^§^ Antibiotics not tested.

**Table 6:** Bacteriological profiling and molecular testing of ESBLs and carbapenemase-producing Gram-negative isolates from patients.

Microbe	Resistant Total *n* (%)	Carbapenem-Resistant Isolates *n* (%)	ESBL ^1^ Isolates *n* (%)
*K. pneumoniae*	135 * (59.7)	115 (85.2)	21 (15.6)
*E. coli*	15 (6.6)	2 (13.3)	13 (86.7)
*P. aeruginosa*	54 (23.9)	28 (51.9)	26 (48.1)
*E. cloacae*	2 (0.9)	1 (50.0)	1 (50.0)
*P. mirabilis*	10 (4.4)	2 (20.0)	8 (80.0)
*S. marcescens*	3 (1.3)	2 (66.7)	1 (33.3)
*M. morganii*	1 (0.4)	1 (100)	- (-)
*P. stuartii*	3 (1.3)	1 (33.3)	2 (66.7)
*A. baumannii*	3 (1.3)	2 (66.7)	1 (33.3)
Total	226	154	73

^1^ ESBL—Extended Spectrum β-Lactamase; * One isolate of *K. pneumoniae* is resistant to both carbapenems and ESBL.

**Table 7:** Carbapenem-resistant isolates.

Microbe	Isolates (*n*) (%)	KPC (*n*) (%)	IMP (*n*) (%)	VIM (*n*) (%)	NDM (*n*) (%)	OXA (*n*) (%)
*K. pneumoniae*	115 (74.7)	113 (98.2)	1 (0.9)	1 (0.9)	1 (0.9)	1 (0.9)
*E. coli*	2 (1.3)	2 (100)	- (-)	- (-)	- (-)	- (-)
*P. aeruginosa*	28 (18.2)	26 (92.9)	- (-)	2 (7.1)	- (-)	- (-)
*E. cloacae*	1 (0.6)	1 (100)	- (-)	- (-)	- (-)	- (-)
*P. mirabilis*	2 (1.3)	2 (100)	- (-)	- (-)	- (-)	- (-)
*S. marcescens*	2 (1.3)	2 (100)	- (-)	- (-)	- (-)	- (-)
*M. morganii*	1 (0.6)	1 (100)	- (-)	- (-)	- (-)	- (-)
*P. stuartii*	1 (0.6)	1 (100)	- (-)	- (-)	- (-)	- (-)
*A. baumannii*	2 (1.3)	2 (100)	- (-)	- (-)	- (-)	- (-)
Total	154	150	1	3	1	1

## Data Availability

The data presented in this study are available on request from the corresponding author due to ethics and patient privacy concerns.

## References

[1] Laxminarayan R., Van Boeckel T., Frost I., Kariuki S., Khan E.A., Limmathurotsakul D., Larsson D.G.J., Levy-Hara G., Mendelson M., Outterson K. (2020). The Lancet Infectious Diseases Commission on antimicrobial resistance: 6 years later. Lancet Infect. Dis..

[2] Tacconelli E., Carrara E., Savoldi A., Harbarth S., Mendelson M., Monnet D.L., Pulcini C., Kahlmeter G., Kluytmans J., Carmeli Y. (2018). Discovery, research, and development of new antibiotics: The WHO priority list of antibiotic-resistant bacteria and tuberculosis. Lancet Infect. Dis..

[3] World Health Organization Ten Threats to Global Health. https://www.who.int/news-room/spotlight/ten-threats-to-global-health-in-2019.

[4] Ahmad M., Khan A.U. (2019). Global economic impact of antibiotic resistance: A review. J. Glob. Antimicrob. Resist..

[5] Marchaim D., Zaidenstein R., Lazarovitch T., Karpuch Y., Ziv T., Weinberger M. (2008). Epidemiology of bacteremia episodes in a single center: Increase in Gram-negative isolates, antibiotics resistance, and patient age. Eur. J. Clin. Microbiol. Infect. Dis..

[6] O’Neill J. Tackling Drug-Resistant Infections Globally: Final Report and Recommendations. The Review on Antimicrobial Resistance, London, 2016. https://apo.org.au/sites/default/files/resource-files/2016-05/apo-nid63983.pdf.

[7] Salam M.A., Al-Amin M.Y., Salam M.T., Pawar J.S., Akhter N., Rabaan A.A., Alqumber M.A.A. (2023). Antimicrobial Resistance: A Growing Serious Threat for Global Public Health. Healthcare.

[8] Kaur N.K.A., Singh S., Singh S. (2017). Prevalence of ESBL and MBL producing gram-negative isolates from various clinical samples in a tertiary care hospital. Int. J. Curr. Microbiol. Appl. Sci..

[9] World Health Organization (2024). WHO Bacterial Priority Pathogens List, 2024: Bacterial Pathogens of Public Health Importance to Guide Research, Development and Strategies to Prevent and Control Antimicrobial Resistance.

[10] World Health Organization WHO Publishes a List of Bacteria for Which New Antibiotics Are Urgently Needed. https://www.who.int/en/news-room/detail/27-02-2017-who-publishes-list-of-bacteria-for-which-new-antibiotics-are-urgently-needed.

[11] Ruppé É., Woerther P.L., Barbier F. (2015). Mechanisms of antimicrobial resistance in Gram-negative bacilli. Ann. Intensive Care.

[12] Bakleh M.Z., Kohailan M., Marwan M., Alhaj Sulaiman A. (2024). A Systematic Review and Comprehensive Analysis of mcr Gene Prevalence in Bacterial Isolates in Arab Countries. Antibiotics.

[13] Codjoe F.S., Donkor E.S. (2017). Carbapenem Resistance: A Review. Med. Sci..

[14] European Centre for Disease Prevention and Control. ECDC, 2023. https://www.ecdc.europa.eu/sites/default/files/documents/healthcare-associated-point-prevalence-survey-acute-care-hospitals-2022-2023.pdf.

[15] European Centre for Disease Prevention and Control (2017). ECDC Country Visit to Italy to Discuss Antimicrobial Resistance Issues. https://www.ecdc.europa.eu/en/publications-data/ecdc-country-visit-italy-discuss-antimicrobial-resistance-issues.

[16] Ashiru-Oredope D., Hopkins S., Vasandani S., Umoh E., Oloyede O., Nilsson A., Kinsman J., Elsert L., Monnet D.L., The #ECDC Antibiotic Survey Project Advisory Group (2021). Healthcare workers’ knowledge, attitudes and behaviours with respect to antibiotics, antibiotic use and antibiotic resistance across 30 EU/EEA countries in 2019. Euro Surveill..

[17] Minogue T.D., Daligault H.A., Davenport K.W., Bishop-Lilly K.A., Broomall S.M., Bruce D.C., Chain P.S., Chertkov O., Coyne S.R., Freitas T. (2014). Complete Genome Assembly of Escherichia coli ATCC 25922, a Serotype O6 Reference Strain. Genome Announc..

[18] European Committee on Antimicrobial Susceptibility Testing Breakpoint Tables for Interpretation of MICs and Zone Diameters Version 15.0, valid from 2025-01-01. https://www.eucast.org/fileadmin/src/media/PDFs/EUCAST_files/Breakpoint_tables/v_15.0_Breakpoint_Tables.pdf.

[19] Magiorakos A.P., Srinivasan A., Carey R.B., Carmeli Y., Falagas M.E., Giske C.G., Harbarth S., Hindler J.F., Kahlmeter G., Olsson-Liljequist B. (2012). Multidrug-resistant, extensively drug-resistant and pandrug-resistant bacteria: An international expert proposal for interim standard definitions for acquired resistance. Clin. Microbiol. Infect..

[20] Morales L., Cobo A., Frías M.P., Gálvez A., Ortega E. (2024). The Prevalence of Antibiotic Resistance Phenotypes and Genotypes in Multidrug-Resistant Bacterial Isolates from the Academic Hospital of Jaén, Spain. Antibiotics.

[21] Vom Steeg L.G., Klein S.L. (2016). SeXX matters in infectious disease pathogenesis. PLoS Pathog..

[22] Onorato L., Sarnelli B., D’Agostino F., Signoriello G., Trama U., D’Argenzio A., Montemurro M.V., Coppola N. (2022). Epidemiological, Clinical and Microbiological Characteristics of Patients with Bloodstream Infections Due to Carbapenem-Resistant *K. Pneumoniae* in Southern Italy: A Multicentre Study. Antibiotics.

[23] Klein S.L., Flanagan K.L. (2016). Sex differences in immune responses. Nat. Rev..

[24] Dias S.P., Brouwer M.C., van de Beek D. (2022). Sex and Gender Differences in Bacterial Infections. Infect. Immun..

[25] Kumwenda P., Adukwu E.C., Tabe E.S., Ujor V.C., Kamudumuli P.S., Ngwira M., Wu J.T.S., Chisale M.R.O. (2021). Prevalence, distribution and antimicrobial susceptibility pattern of bacterial isolates from a tertiary Hospital in Malawi. BMC Infect. Dis..

[26] Hammour K.A., Abu-Farha R., Itani R., Karout S., Allan A., Manaseer Q., Hammour W.A. (2023). The prevalence of Carbapenem Resistance Gram negative pathogens in a Tertiary Teaching Hospital in Jordan. BMC Infect. Dis..

[27] Monnet D.L., Harbarth S. (2020). Will coronavirus disease (COVID-19) have an impact on antimicrobial resistance?. Euro Surveill..

[28] Rawson T.M., Ming D., Ahmad R., Moore L.S.P., Holmes A.H. (2020). Antimicrobial use, drug-resistant infections and COVID-19. Nat. Rev. Microbiol..

[29] Musicha P., Cornick J.E., Bar-Zeev N., French N., Masesa C., Denis B., Kennedy N., Mallewa J., Gordon M.A., Msefula C.L. (2017). Trends in antimicrobial resistance in bloodstream infection isolates at a large urban hospital in Malawi (1998–2016): A surveillance study. Lancet Infect. Dis..

[30] Iroh Tam P.Y., Musicha P., Kawaza K., Cornick J., Denis B., Freyne B., Everett D., Dube Q., French N., Feasey N. (2019). Emerging Resistance to Empiric Antimicrobial Regimens for Pediatric Bloodstream Infections in Malawi (1998–2017). Clin. Infect. Dis..

[31] Goebel M.C., Trautner B.W., Grigoryan L. (2021). The Five Ds of Outpatient Antibiotic Stewardship for Urinary Tract Infections. Clin. Microbiol. Rev..

[32] Iacchini S., Boros S., Pezzotti P., Caramia A., Errico E., Del Grosso M., Camilli R., Giufrè M., Pantosti A., Maraglino F. AR-ISS: Sorveglianza Nazionale dell’Antibiotico-Resistenza. Dati 2022. Rapporti ISS Sorveglianza RIS-4/2023. https://www.epicentro.iss.it/antibiotico-resistenza/ar-iss/RIS-4_2023.pdf.

[33] Rodriguez-Maresca M., Sorlozano A., Grau M., Rodriguez-Castado R., Rutz-Valverde A., Gutierrez-Fernandez J. (2014). Implementation of a computerized decision support system to improve the appropriateness of antibiotic therapy using local microbiologic data. Biomed. Res. Int..

[34] Mangioni D., Viaggi B., Giani T., Arena F., D’Arienzo S., Forni S., Tulli G., Rossolini G.M. (2019). Diagnostic stewardship for sepsis: The need for risk stratification to triage patients for fast microbiology workflows. Future Microbiol..

[35] Walker T., Dumadag S., Jiyoun Lee C., Lee S.H., Bender J.M., Cupo Abbott J., She R.C. (2016). Clinical impact of laboratory implementation of Verigene BC-GN microarray-based assay for detection of Gram-negative bacteria in positive blood cultures. J. Clin. Microbiol..

[36] Ferri M., Ranucci E., Romagnoli P., Giaccone V. (2017). Antimicrobial resistance: A global emerging threat to public health systems. Crit. Rev. Food Sci. Nutr..

